# Providing an interactive undergraduate elective on safety culture online – concept and evaluation

**DOI:** 10.1186/s12909-022-03541-1

**Published:** 2022-06-28

**Authors:** Angelina Müller, Olga A. Sawicki, Marina Pommee, Tatjana Blazejewski, Kyra Schneider, Judith Ullmann-Moskovits, Juliana J. Petersen, Beate S. Müller

**Affiliations:** 1 Institute of General Practice, Goethe University Frankfurt, Theodor-Stern-Kai 7, 60590 Frankfurt, Germany; 2 Stabsstelle Patientensicherheit & Qualität, Universitätsklinikum Frankfurt, Theodor-Stern-Kai 7, 60590 Frankfurt, Germany

**Keywords:** Safety culture, Online education, Interaction, Undergraduate, Patient safety

## Abstract

**Background:**

The COVID-19 pandemic has made it more difficult to maintain high quality in medical education. As online formats are often considered unsuitable, interactive workshops and seminars have particularly often been postponed or cancelled. To meet the challenge, we converted an existing interactive undergraduate elective on safety culture into an online event. In this article, we describe the conceptualization and evaluation of the elective.

**Methods:**

The learning objectives of the safety culture elective remained unchanged, but the teaching methods were thoroughly revised and adapted to suit an online setting. The online elective was offered as a synchronous two-day course in winter semester 2020/21 during the “second wave” of the COVID-19 pandemic in Germany. At the end of each day, participating students evaluated the elective by completing an online survey. Items were rated on a six-point Likert scale. We used SPSS for data analysis.

**Results:**

Twenty medical undergraduates completed the elective and rated it extremely positively (1.1 ± 0.2). Students regard safety culture as very important and felt the learning objectives had been achieved. Moreover, they were very satisfied with the design and content of the elective, and especially with interactive elements like role-play. Around 55% of participants would recommend continuing to offer the online elective after the pandemic.

**Conclusions:**

It makes sense to offer undergraduate medical students online elective courses on safety culture, especially during a pandemic. The elective described here can serve as a best practice example of how to teach safety culture to undergraduates, especially when physical presence is unfeasible. Electives requiring a high degree of interaction can also function well online.

**Supplementary Information:**

The online version contains supplementary material available at 10.1186/s12909-022-03541-1.

## Background

During the COVID-19 pandemic, medical education had to be adapted to the new situation. Worldwide, assessments and placements had to be cancelled, postponed or offered in a different format [1]. A review of the immediate consequences of the pandemic for the education of medical students and residents revealed that the most common change was to switch to an online format [2]. Among online courses, presentations, virtual case reviews and tutorials were commonly used [2]. The authors of the review also stated that reports of a switch to online courses for medical students understate the true situation [2]. The transition to an online implementation was found throughout the entire preclinical setting [3]. However, online courses rarely employ multimedia design principles in undergraduate medical education [4].

Medical electives have proved themselves to be beneficial to the individual development of future physicians [5] and help medical students acquire useful skills outside the traditional curriculum [6]. Electives on safety culture are a valuable means of imparting knowledge on the subject, especially in view of World Health Organization (WHO) recommendations that the profile of patient safety in medical education should be raised [7].

In response to the WHO recommendation, several universities in Germany are now offering courses on safety culture [8], mostly as part of the advanced medical curriculum. We explicitly chose to offer the elective to undergraduates and thus to follow recommendations to teach the subject at an early stage of medical studies [7].

Medical students require intensive coaching in soft skills, such as communication skills [9], and in dealing with medical errors and safety culture in general [7]. Role-play and simulations are suitable techniques to train these skills [10]. Despite COVID-19-related contact restrictions, we wanted to avoid leaving out these interactive elements, as confining teaching to theoretical aspects would negatively impact the skill-sets of future physicians [11, 12].

It was necessary to develop a robust online concept, as opposed to simply using flipped classrooms and recording lectures [13]. This is especially true for sensitive topics such as safety culture. We therefore developed an online elective on safety culture for undergraduate medical students that was based on a previously described and evaluated in-person elective [8]. We retained elements that successfully involved a high degree of interaction such as role-play, and modified them according to multimedia design principles from the Association of American Medical Colleges Institute on the Effective Use of Educational Technology in Medical Education [14], as these have been shown to improve knowledge retention in medical education [15]. This article describes the development and evaluation of a two-day synchronous online elective for undergraduate medical students.

## Methods

### Concept

The safety culture elective was originally conceptualized by an interprofessional team of authors (physicians, health scientists, a psychologist and a bioscientist) and developed by the following lecturers, AM, OAS, MP, TB, KS and BSM. The learning objectives were based on the National Competence-Based Learning Goal Catalogue for Medical Studies (NKLM) [16]. The principal learning objectives were concerned with safety culture and patient safety, as well as communication when an error has occurred. All learning objectives are displayed in Table 1.

**Table 1:** Learning objectives

Students were required to…
… explain various influencing factors that may lead to the development of complications.
… based on an existing medical narrative involving a critical incident, develop an incident report and detail possible consequences.
… reflect upon their own and others ‘ conduct, identify errors and discuss these appropriately with colleagues and supervisors.
… explain the importance of a safety culture when reporting on critical incidents and learning from them.
… know the most important aspects of complication management, risk communication, Critical Incident Reporting System (CIRS), and recognizing critical incidents, and have been taught how to deal with wrong decisions.

We based the development of this online elective on our own preparatory work on the topic of safety culture, as well as our teaching experience, including our medical-didactic knowledge of online formats [17–19]. The formal requirement for the undergraduate elective was the approval of the curriculum by the study commission. This was obtained previously due to importance of the topic. The organizational framework set forth by the Department of Medicine was two hours a week for one semester (16 h). We divided the 16 h into two block seminars of 8 h in length, conducted as synchronous video conference using the software Zoom on two consecutive days in the winter semester of 2020/21. Final grades were based on active participation during the elective and the assessment of a Critical Incident Reporting System (CIRS) report.

The online elective on safety culture was developed on the basis of the in-person elective we offered in 2020 [8]. Established multimedia design principles [14] were used when showing presentations. Since the elective was mainly carried out synchronously, we focused on in-person didactical principles in the interactive parts.

### Participants

It was possible for a total of 20 medical students in their third undergraduate semester to voluntarily enrol in the online elective.

### The online elective on safety culture

The structure of the online elective is presented in Table 2.

**Table 2:** The online elective on safety culture

Module / Content	Method	Video conference feature/Multimedia usage	Length [min]
Day 1			
Introduction, agenda, atmosphere, energy levels, introduction of team, participants introduce themselves	Frontal instruction and moderated group discussion using PowerPoint	Screen sharing (PowerPoint-Slides)	30
Previous experience of medical errors or risks? Reflection in the group on own experience of error, risks, safety culture.		Discussion in break-out sessions, each involving four participants (15 min.). One person from each group reports on the group’s discussions/experiences	45
What is an error? German Coalition for Patient Safety – Error: " An action or omission that entails deviating from the plan, following a wrong plan, or no plan. Whether harm arises from this is irrelevant for the definition of an error."	First let individuals think for themselves (5 min), then gather results on a flipchart (5 min)	Digital flipchart	15
Types of errors and incidents	Lecture	Screen sharing (PowerPoint-Slides)	15
Example of interprofessional communication failure German Film "Everything humanly possible", min. 5:37–15:42. (Story in short: busy emergency department, malfunctioning interprofessional communication, sepsis patient dies due to a confusion)	Watch the film clip individually	Cover camera with piece of paper	20
Reflection on film example (I): How do I feel? What, in my view, contributed to the event? Who is “responsible” for the death of the patient? (note: in discussion make clear that “responsibility” should not be the question here but how to prevent incidents from happening again and how to reduce risks)	Participants take different perspectives and reflect on questions (perspectives: female doctor, patient’s husband, female nurse)	Discussion in 6 breakout sessions (2 for each perspective) with 3–4 participants (15 min.) and subsequently in the whole group (15 min.)	30
Explanation of the term "second victim"	Lecture	Screen sharing (PowerPoint-Slides)	5
Short break			5
Theoretical input: - Why should we talk about errors, incidents, risks? - Swiss cheese model - Eisberg model, contributing factors - Human factors vs. system failure		Interactive lecture with screen sharing (PowerPoint-Slides) and with involvement of whole group. Write down why errors occur (each for him/herself and then collect results on a digital flipchart	15
Feedback on the morning (too much, too fast?)			
Lunch break			60
Introductory warm-up	Activation by exercising together, e.g. - Those that ate a warm meal for lunch must walk twice around their chairs! - Those that went outside during the lunchbreak can remain seated. All others do 3 squats. - …		5
Analysis of adverse events: How did it happen?	In the entire group, present and analyze an example that has been described in the media (train accident)	Interactive lecture with screen sharing (PowerPoint-Slides) and with involvement of whole group.	10
Reflection on film example (II): How did the event occur? Watch the film clip again and make notes -When did something go wrong? -What factors contributed (write down contributing factors)?	Watch the film clip asynchronously and make individual notes. Afterwards, gather notes orally in the whole group		30
Energy levels? Short coffee break?			
Levels of preventive measures (weak, moderate, strong) Principle of strong preventive measures ("The system must ensure it is difficult to make an error."). Leadership involvement as strong measure.	Lecture	Screen sharing (PowerPoint-Slides)	10
Reflection on film example (III): Develop preventive measures (error prevention and risk reduction) Which of the measures might prevent the next patient with sepsis from dying?		First think of one preventive measure for each level, then present these measures in breakout sessions with 3–4 persons, decide on two for each level (weak, moderate, strong) and present them to the whole group	30
Feedback and evaluation day 1		In the entire group, evaluation via online link	5
Work up an incident report from the CIRS www.jeder-fehler-zaehlt.de (“every error counts”) chronologically, and think of at least one weak, one moderate and one strong preventive measure	Work in small groups of 1–4 persons (the groups can leave the video conference to continue working after they have decided on a report)		150
**Day 2**			
Introduction: What do participants remember from day 1?		Digital flipchart	15
Which CIRS exist in Germany for which target groups? Describe how CIRS work		Interactive lecture with screen sharing (PowerPoint-Slides) Afterwards, each participant seeks web-based CIRS. Subsequently, collect the results and discuss them using a digital flipchart	15
Presentation and discussion of the CIRS cases and preventive measures that were worked on the previous day		4 Breakout sessions. Groups present their cases and preventive measures to each other. In small groups, agree on the best preventive measure for each case	35
Short break			5
How do critical incident reporting and learning systems work? Example: Frankfurt University Hospital’s CIRS		Interactive lecture with screen sharing (PowerPoint-Slides)	90
Preparation of a user comment with a recommendation for a preventive measure on www.jeder-fehler-zaehlt.de	Each group on its own		15
Lunch break			60
Ice-breaker: Place a virtual stamp according to desired medical specialty (part of the body) and preferred place of work (map of Germany)		Stamps entered onto PowerPoint slides with the entire group (feature of the video conference system)	15
Feedback rules		Collection on a digital flipchart and discussion	30
Communication about critical incidents, errors, risks What would encourage people to talk about errors and risks? What is important to make people speak up in hierarchical structures? What makes good communication in interdisciplinary teams?	Role-play (4 scenarios in break-out sessions with subsequent feedback / reflection in small groups)—> Inclusion of students ‘ experience of errors.	Break-out sessions	35
Individual responsibility and individual possibilities - Question: What will I do differently tomorrow?		In the whole group, one after the other Reflection	30
Feedback and evaluation day 2		In the entire group, evaluation via online link	10
Any further questions?		In the entire group	5

### Role-play

Role-play was used in break-out sessions, whereby four students were assigned to one lecturer and each group was provided with one scenario. The available roles were those of doctors, their supervisors and colleagues that were involved in a conflict that had to be resolved. The scenarios resulted from a situation in which an error had been made that compromised patient safety. The scenarios included several hierarchical levels and professional groups, so that the role of the (interdisciplinary) leadership in building a strengthened safety culture could be reflected from different perspectives. One student in each group acted as an observer and was responsible for providing feedback to the other students. The lecturers also gave constructive feedback.

### Evaluation

We used a standardized online questionnaire to evaluate the elective. The questionnaire was based on the existing evaluation templates of the Institute of General Practice and the Department of Medicine [20]. It consisted of 17 items to be completed on the first day and 18 items to be completed on the second. The original German version as well as the English translation are available in the supplement. An online link was used to access the questionnaire and participants filled it out anonymously.

Most items were rated on a six-point Likert scale, with the points equivalent to German school grades (1 = very good, 6 = insufficient). The participants also had the opportunity to comment on strengths and limitations, as well as any need for improvement, in the online elective in free-form text. We expressly asked the students to comment on strengths and any need for improvement.

### Statistical analysis

We used descriptive statistics to analyze the results. Arithmetic means and standard deviations of the values are presented below. IBM SPSS Statistics 25 software was used for data analysis.

## Results

The 20 available places in the online elective were all taken up. Of the 20–30 year old participants, 80% were female and 20% male, while 20% had previous experience in the field of medicine, e.g. as practical trainees, research assistants or paramedics. The evaluation results are presented in Table 3.

**Table 3:** Evaluation results

	**Possible responses in % (*N* = 20)** **1 = Completely agree, 6 = Completely disagree after day 1/ after day 2**						**Mean (standard deviation)**
	**1**	**2**	**3**	**4**	**5**	**6**	
**I consider the addressed topics to be important. **^**a**^	80.0/70.0	20.0/15.0	0.0/5.0	0.0/0.0	0.0/0.0	0.0/0.0	1.2(0.4)/1.5 (0.8)
**The content of the seminar was well structured. **^**a**^	95.0/65.0	5.0/30.0	0.0/5.0	0.0/0.0	0.0/0.0	0.0/0.0	1.1(0.2)/1.4(0.6)
**The content was presented in a comprehensible manner. **^**a**^	100.0/85.0	0.0/15.0	0.0/0.0	0.0/0.0	0.0/0.0	0.0/0.0	1.0(0.0)/1.2(0.4)
**The amount of material was appropriate. **^**a**^	80.0/80.0	15.0/15.0	5.0/5.0	0.0/0.0	0.0/0.0	0.0/0.0	1.3(0.6)/1.3(0.6)
**I could participate actively. **^**a**^	100.0/85.0	0.0/10.0	0.0/5.0	0.0/0.0	0.0/0.0	0.0/0.0	1.0(0.0)/1.2(0.5)
**I learned something from the seminar. **^**a**^	45.0/70.0	50.0/25.0	5.0/5.0	0.0/0.0	0.0/0.0	0.0/0.0	1.6(0.6)/1.4(0.6)
**The employed teaching methods (digital whiteboard, breakout-sessions) helped convey the content. **^**a**^	70.0/80.0	30.0/15.0	0.0/5.0	0.0/0.0	0.0/0.0	0.0/0.0	1.3(0.5)/1.3(0.6)
**Role-play…**							
** …enabled learning content to be conveyed well**	70.0	20.0	5.0	0.0	0.0	0.0	1.5(1.0)
** … enabled the topic to be dealt with comprehensibly and with practical relevance**	70.0	20.0	5.0	0.0	0.0	0.0	1.5(1.0)
** I would recommend the seminar to other students**	100.0	0.0	0.0	0.0	0.0	0.0	1.0(0.0)
** This elective should continue to be provided in an online format after Corona**	55.0	5.0	30.0	0.0	0.0	0.0	2.0(1.3)
** Overall assessment of the seminar**	95.0	5.0	0.0	0.0	0.0	0.0	1.1(0.2)

^a^ after day 1/ after day 2

The response rate was 100% (20/20). The lecturers were rated by the students as highly motivated (mean 1.1, standard deviation (SD) 0.0) and good at teaching (mean 1.3; SD 0.4). The students also rated the lecturers’ ability to actively involve them in the course positively (mean 1.1; SD 0.2).

All students gave additional feedback voluntarily. They considered the structure and teaching atmosphere of the elective to be reassuring. Furthermore, the high levels of interactivity within the group were remarked upon. Students explicitly mentioned that working on group tasks in break-out sessions and participating in role-play helped them achieve their learning objectives. They did not consider the virtual design to have impaired their assessment of safety culture topics. The following summaries are examples of answers to the question what students particularly liked about the elective:

*"The learning atmosphere was very pleasant throughout the group, right from the beginning, and that helped keep any inhibitions to participating actively to a minimum. Furthermore, the variety, with short presentations, discussions in small groups and in the whole group as well, was very good because it stopped things becoming boring and monotonous"*

*"The content of the seminar was very important and interesting. Theoretical aspects were communicated interactively and in a good way, so you never felt bored. I also thought the break-out sessions were particularly practical because swapping more personal information is probably easier in a smaller group. Overall, a great success!"*

*"The interaction; lots of different impressions; the best possible implementation of the course despite limited possibilities (Corona zoom); the feedback session that followed the role-play on help through self-reflection."*

*"Looking for possible solutions yourself; using role-play to creatively apply what you have learned, and a very good atmosphere within the group."*

Suggestions for improvement included more short and scheduled breaks of three to five minutes. With respect to content, students would have preferred more psychological background information. One student suggested employing role-play at the beginning and the end of the elective to assess the effectiveness of the training.

## Discussion

The adoption of an online format for undergraduate courses on safety culture would appear to be a sensible reaction to the COVID-19 pandemic. Students were very satisfied with the design and content of the elective, and particularly with the opportunity for intense interaction provided by role-play.

Safety culture and patient safety face enhanced challenges in times of COVID-19 [21, 22]. As a result of the pandemic, both dealing with errors professionally and communicating effectively are becoming increasingly important and may help protect patients in the future. It is essential to maintain high quality in medical education with respect to safety culture and communication and to raise students’ awareness of the importance of good interdisciplinary teamwork across hierarchical levels.

### Effectiveness

After finding no differences in learning outcomes, one review has suggested that online education is as effective as traditional teaching, [23]. We successfully used online teaching techniques such as defining tasks and discussing them online [24]. A U.S. study has described an online elective for undergraduates [25] that involved virtual students interacting with patients. Since the communication strategies were the same as for real students, the online elective also succeeded in training communication skills.

### Limitations and strengths

As the number of participants was limited to 20 persons, the size of our sample was small. Since students enrolled in the elective voluntarily and probably had a previous interest in the topic, a self-selection bias cannot be ruled out, and they may have been more likely to be satisfied with the content. However, this effect was unlikely to have had a significant influence on perceptions of the online format. It is also worthy of note that one participant considered the use of diverse techniques to have enhanced the elective. However, as no other course in the preceding 10 months of the pandemic had yet used them, it was impossible to compare our use of new techniques with those of others. Furthermore, social distancing (no classes with colleagues for months) may have increased the readiness of the participants to be content with the elective, simply because they welcomed the chance to solve tasks and learn in groups again. The circumstances and conditions may therefore have had a greater effect on their assessments than learning outcomes [4]. Moreover, undergraduate students are more likely to rate online classes positively than students in clinical semesters [26]. As we have not conducted a formal examination, we cannot assess whether participants have achieved the learning objectives. This would be a valuable addition to a future elective.

Furthermore, the study was based on a single elective at a single academic institution. The authors would therefore recommend that lecturers consider employing both asynchronous and synchronous online teaching opportunities [27] in order to balance the pros and cons for specific users [28]. A limitation of our synchronous format is that it leaves the audience little time to reflect on the topic. We attempted to implement asynchronous elements by reflecting on the homework each student had to do between day 1 and day 2. As students also had the opportunity to give feedback at any time, we were also able to change the pace of instruction in line with their individual wishes.

One strength of our synchronous concept is that after some preparation, online interactions could promote group discussion, encourage social interaction and enable participants to plan tasks that required real-time feedback [27]. All these elements were included in our online elective.

## Conclusions

The use of an online elective to educate undergraduate medical students in patient safety is a feasible alternative to classroom instruction. In medical education, interactive elements can be implemented in various ways, and include training for doctor-patient consultations. Insights from our study can therefore be transferred to various settings in which medical education is maintained despite the pandemic. Electives with interactive elements, even those of high intensity, can be successfully designed using an online format. This elective can serve as a best practice example of how to use a multimedia design to teach patient safety. Although the application of the science of learning to medical education is widely recommended [15, 29], we would like to point out that online courses require elaborate preparation that is adapted to suit the needs of an online format [27, 28].

## Supplementary Information

**Additional file 1.**

## Data Availability

All data generated or analysed during this study are included in this published article [and its supplementary information files]. Any further information can be provided by the corresponding author upon request.

## Abbreviations

CIRS: Critical incident reporting system
NKLM: National Competence-Based Learning Goal Catalogue for Medical Studies
SD: Standard deviation
WHO: World Health Organization
