# Supplementary material for: Providing an interactive undergraduate elective on safety culture online – concept and evaluation
Source: BMC Med Educ. 2022 Jun 28;22:508. doi: 10.1186/s12909-022-03541-1 (PMC9238086; doi:10.1186/s12909-022-03541-1)
Supplement: Supplementary file 1 — Additional file 1. [file 12909_2022_3541_MOESM1_ESM.zip › Fragebogen_WS2021_1.pdf]

EvaSys	Wahlpflichtfach Vorklinik „Sicherheitskultur in der Medizin: Jeder Fehler zählt!“	Electric Paper

Bitte so markieren: ☐ ☒ ☐ ☐ ☐ Bitte verwenden Sie einen Kugelschreiber oder nicht zu starken Filzstift. Dieser Fragebogen wird maschinell erfasst.

Korrektur: ☐ ■ ☐ ☒ ☐ Bitte beachten Sie im Interesse einer optimalen Datenerfassung die links gegebenen Hinweise beim Ausfüllen.

Seminarevaluation S1 (14.01.2021) - Wintersemester 2020/2021

Liebe Studierende,

um unser Wahlpflichtfach kontinuierlich zu verbessern, freuen wir uns über Deine Rückmeldung zum heutigen Seminar.

1. Anonymer Personencode

1.1 **Buchstabe des Vornamens Ihrer Mutter**
Beispiel: Helga -> H
Hinweis: Sollte Ihnen die Information nicht bekannt sein, tragen Sie bitte den Buchstaben X ein.

1.2 **Buchstabe des ersten Studienortes, an dem Sie Medizin studierten**
Beispiel: Leipzig -> L

1.3 **Geburtstag der Mutter** (**TT**.MM.JJJJ)
Hinweis: Sollte Ihnen die Information nicht bekannt sein, tragen Sie bitte die Ziffern 00 ein.

1.4 **Buchstabe Ihres Geburtortes**
Beispiel: Bad Hersfeld -> B

2. Personenbezogene Angaben

2.1 Geburtsjahr

2.2 Bitte geben Sie Ihr Geschlecht an. ☐ männlich ☐ weiblich ☐ inter/non-binär ☐ keine Angabe

2. Personenbezogene Angaben [Fortsetzung]

2.3 Falls vorhanden, Berufserfahrung in einem medizinischen Beruf:

2.4 Ich kann mir vorstellen, Hausarzt/Hausärztin zu werden Triff voll zu ☐ ☐ ☐ ☐ ☐ ☐ Trifft gar nicht zu

Wie beurteilst Du folgende Aussagen bezüglich des heutigen Seminars?

3. Inhalt

3.1 Die angesprochenen Themen waren für mich relevant. Trifft voll zu ☐ ☐ ☐ ☐ ☐ ☐ Trifft gar nicht zu

3.2 Der inhaltliche Aufbau des Seminars war gut. Trifft voll zu ☐ ☐ ☐ ☐ ☐ ☐ Trifft gar nicht zu

4. Didaktik

4.1 Der Inhalt wurde verständlich dargestellt. Trifft voll zu ☐ ☐ ☐ ☐ ☐ ☐ Trifft gar nicht zu

4.2 Die Stoffmenge war angemessen. Trifft voll zu ☐ ☐ ☐ ☐ ☐ ☐ Trifft gar nicht zu

4.3 Ich konnte mich aktiv beteiligen. Trifft voll zu ☐ ☐ ☐ ☐ ☐ ☐ Trifft gar nicht zu

4.4 Ich habe durch das Seminar einen Lernzuwachs erfahren. Trifft voll zu ☐ ☐ ☐ ☐ ☐ ☐ Trifft gar nicht zu

4.5 Die eingesetzten Medien (Zoom, Whiteboard, Break-Out-Sessions) haben die Vermittlung der Inhalte unterstützt. Trifft voll zu ☐ ☐ ☐ ☐ ☐ ☐ Trifft gar nicht zu

5. Die Dozentinnen ...

5.1 ... haben die Lerninhalte gut vermittelt. Trifft voll zu ☐ ☐ ☐ ☐ ☐ ☐ Trifft gar nicht zu

5.2 ... waren engagiert. Trifft voll zu ☐ ☐ ☐ ☐ ☐ ☐ Trifft gar nicht zu

5.3 ... haben die Studierenden gut eingebunden. Trifft voll zu ☐ ☐ ☐ ☐ ☐ ☐ Trifft gar nicht zu

6. Kommentare

6.1 Das hat mir am heutigen Seminar besonders gut gefallen ...

6.2 Das könnte man noch verbessern ...
