# Supplementary material for: Providing an interactive undergraduate elective on safety culture online – concept and evaluation
Source: BMC Med Educ. 2022 Jun 28;22:508. doi: 10.1186/s12909-022-03541-1 (PMC9238086; doi:10.1186/s12909-022-03541-1)
Supplement: Supplementary file 1 — Additional file 1. [file 12909_2022_3541_MOESM1_ESM.zip › Fragebogen_WS2021_2.pdf]

EvaSys	Wahlpflichtfach Vorklinik „Sicherheitskultur in der Medizin: Jeder Fehler zählt!“	Electric Paper

Bitte so markieren: ☐ ☒ ☐ ☐ ☐ Bitte verwenden Sie einen Kugelschreiber oder nicht zu starken Filzstift. Dieser Fragebogen wird maschinell erfasst.

Korrektur: ☐ ■ ☐ ☒ ☐ Bitte beachten Sie im Interesse einer optimalen Datenerfassung die links gegebenen Hinweise beim Ausfüllen.

Seminarevaluation S2 (15.01.2021) - Wintersemester 2020/2021

Liebe Studierende,

um unser Wahlpflichtfach kontinuierlich zu verbessern, freuen wir uns über Deine Rückmeldung zum heutigen Seminar.

1. Anonymer Personencode

1.1 **Buchstabe des Vornamens Ihrer Mutter**
Beispiel: Helga -> H
Hinweis: Sollte Ihnen die Information nicht bekannt sein, tragen Sie bitte den Buchstaben X ein.

1.2 **Buchstabe des ersten Studienortes, an dem Sie Medizin studierten**
Beispiel: Leipzig -> L

1.3 **Geburtstag der Mutter** (**TT**.MM.JJJJ)
Hinweis: Sollte Ihnen die Information nicht bekannt sein, tragen Sie bitte die Ziffern 00 ein.

1.4 **Buchstabe Ihres Geburtortes**
Beispiel: Bad Hersfeld -> B

Wie beurteilst Du folgende Aussagen bezüglich des heutigen Seminars?

2. Inhalt

2.1	Die angesprochenen Themen waren für mich relevant.	Trifft voll zu	☐	☐	☐	☐	☐	☐	Trifft gar nicht zu
2.2	Der inhaltliche Aufbau des Seminars war gut.	Trifft voll zu	☐	☐	☐	☐	☐	☐	Trifft gar nicht zu

3. Didaktik

3.1	Der Inhalt wurde verständlich dargestellt.	Trifft voll zu	☐	☐	☐	☐	☐	☐	Trifft gar nicht zu
3.2	Die Stoffmenge war angemessen.	Trifft voll zu	☐	☐	☐	☐	☐	☐	Trifft gar nicht zu
3.3	Ich konnte mich aktiv beteiligen.	Trifft voll zu	☐	☐	☐	☐	☐	☐	Trifft gar nicht zu
3.4	Ich habe durch das Seminar einen Lernzuwachs erfahren.	Trifft voll zu	☐	☐	☐	☐	☐	☐	Trifft gar nicht zu
3.5	Die eingesetzten Medien (Zoom, Whiteboard, Break-Out-Sessions) haben die Vermittlung der Inhalte unterstützt.	Trifft voll zu	☐	☐	☐	☐	☐	☐	Trifft gar nicht zu

4. Durch Rollenspiele ...

4.1	... wurden die Lerninhalte gut vermittelt.	Trifft voll zu	☐	☐	☐	☐	☐	☐	Trifft gar nicht zu
4.2	... wurde das Thema verständlich und praxisnah aufgearbeitet.	Trifft voll zu	☐	☐	☐	☐	☐	☐	Trifft gar nicht zu

5. Die Dozentinnen ...

5.1	... haben die Lerninhalte gut vermittelt.	Trifft voll zu	☐	☐	☐	☐	☐	☐	Trifft gar nicht zu
5.2	... waren engagiert.	Trifft voll zu	☐	☐	☐	☐	☐	☐	Trifft gar nicht zu
5.3	... haben die Studierenden gut eingebunden.	Trifft voll zu	☐	☐	☐	☐	☐	☐	Trifft gar nicht zu

6. Kommentare

6.1 Das hat mir am heutigen Seminar besonders gut gefallen ...

6.2 Das könnte man noch verbessern ...

7. Weitere Fragen (beziehen sich auf beide Seminartage)

7. Weitere Fragen (beziehen sich auf beide Seminartage) [Fortsetzung]

7.1	Ich würde das Seminar anderen Studierenden empfehlen.	Trifft voll zu	☐	☐	☐	☐	☐	☐	Trifft gar nicht zu
7.2	Dieses Wahlfach soll auch nach Corona als *Online-Seminar* fortgeführt werden.	Trifft voll zu	☐	☐	☐	☐	☐	☐	Trifft gar nicht zu
7.3	Gesamtbewertung des Seminars (Schulnote 1 bis 6)	1	☐	☐	☐	☐	☐	☐	6
